# The Effects of Microplastics and Trace Elements on Ovarian Function and Pathology: A Literature Review

**DOI:** 10.3390/ijms27188306

**Published:** 2026-09-18

**Authors:** Łukasz Bryliński, Katarzyna Brylińska, Jolanta Sado, Kacper Kraśnik, Miłosz Smyk, Olga Komar, Filip Woliński, Michał Flieger, Jolanta Flieger, Grzegorz Teresiński, Alicja Forma, Jacek Baj

**Affiliations:** 1 Department of Forensic Medicine, Medical University of Lublin, Jaczewskiego 8b, 20-090 Lublin, Poland; michalflieeeger@gmail.com (M.F.); grzegorz.teresinski@umlub.edu.pl (G.T.); 2 Doctoral School, Medical University of Lublin, Chodźki 7, 20-093 Lublin, Poland; 3 Department of Correct, Clinical and Imaging Anatomy, Medical University of Lublin, ul. Jaczewskiego 4, 20-090 Lublin, Polandolgakomar720@gmail.com (O.K.); rush22235@gmail.com (F.W.); alicja.forma@umlub.edu.pl (A.F.); jacek.baj@umlub.edu.pl (J.B.); 4 Student Scientific Society of Correct, Clinical and Imaging Anatomy, Medical University of Lublin, ul. Jaczewskiego 4, 20-090 Lublin, Poland; jjsado@wp.pl (J.S.); kacper.krasnik@gmail.com (K.K.); miloszs.105@gmail.com (M.S.); 5 Department of Analytical Chemistry, Medical University of Lublin, Chodźki 41 (Collegium Pharmaceutcum), 20-093 Lublin, Poland; jolanta.flieger@umlub.edu.pl

**Keywords:** nanoplastics, ovary, polycystic ovary syndrome, gonadal dysfunction, infertility, oxidative stress, cellular toxicity

## Abstract

The female reproductive system, particularly the ovary, exhibits a high sensitivity to the effects of environmental xenobiotics. This group of substances includes micro- and nanoplastics (MNPs) as well as trace elements. This literature review summarises the current knowledge on the individual and combined effects of MNPs and trace elements—including essential bioelements (zinc (Zn), selenium (Se), copper (Cu), chromium (Cr), manganese (Mn)) and toxic elements (cadmium (Cd), Lead (Pb), arsenic (As), mercury (Hg))—on ovarian physiology and clinical conditions such as polycystic ovary syndrome (PCOS), gonadal dysfunction and infertility. Essential trace elements play irreplaceable roles as enzyme cofactors (e.g., superoxide dismutase (SOD), glutathione peroxidase (GPX)) and transcriptional regulators; however, their biological action is confined to a narrow homeostatic window, within which both deficiency and excess disrupt cellular redox status and the hypothalamic–pituitary –gonadal axis. The effect of toxic heavy metals is different; these exhibit threshold-independent cytotoxic, genotoxic and endocrine-disrupting effects, inducing apoptosis of granulosa cells, endoplasmic reticulum stress and the silencing of steroidogenic genes (e.g., *Steroidogenic acute regulatory protein (StAR)*, *CYP11A1*, *CYP19A1*) via epigenetic modifications, which accelerates the depletion of the ovarian reserve (measured by a decline in anti-Müllerian hormone (AMH)). Concurrently, MNPs overcome biological barriers and accumulate within the ovarian microenvironment, thereby inducing reactive oxygen species (ROS)-dependent apoptosis, NLR family pyrin domain-containing 3 (NLRP3) inflammasome-mediated pyroptosis, cytoskeletal disruption, and tissue fibrosis. A key element of this review is the identification of mechanistic convergence and potential toxic synergism between MNPs and heavy metals. Co-exposure enhances toxin retention within cells by inhibiting ATP-binding cassette (ABC) transporters, potentiates mitochondrial depolarisation, and activates protein kinase RNA-like endoplasmic reticulum kinase–eukaryotic translation initiation factor 2 alpha (PERK/eIF2α) stress signalling. Understanding these shared molecular pathways forms the basis for assessing multi-component risk in female reproductive toxicology and for developing targeted protective strategies.

## 1. Introduction

Typically situated in the ovarian fossa, at the bifurcation of the external and internal iliac arteries, the ovary is a paired, intraperitoneal exocrine organ measuring, on average, 2.0 cm in width, 3.5 cm in length and 1.0 cm in thickness [1]. Anatomically, this organ consists of three zones: the cortex, the medulla and the lumen. Because of their structure, which consists of specialised cells, the ovaries perform both endocrine and reproductive functions. Their endocrine function involves the production of sex hormones, such as progesterone and oestrogens, which play important roles in reproductive functions and in the proper functioning and metabolism of the entire body [2,3]. Furthermore, thanks to its cellular structure, which contains oocytes at various developmental stages, this organ is involved in the production of gametes that undergo fertilisation [4].

Many factors influence the normal function of the ovary. The most important of these is the hypothalamic–pituitary–gonadal (HPG) axis, the primary neuroendocrine pathway. GnRH, produced by the hypothalamus, stimulates the pituitary gland to secrete two hormones: luteinising hormone (LH) and follicle-stimulating hormone (FSH). These, in turn, regulate gonadal steroidogenesis and gametogenesis [5]. Other factors influencing ovarian function include: a healthy diet and lifestyle, other hormonal disorders such as insulin resistance and hyperinsulinaemia; smoking; and exposure to chemical substances such as bisphenols. The adverse effects of these factors may lead to impaired ovarian function and the development of conditions such as polycystic ovary syndrome (PCOS), ovarian dysfunction and infertility [6,7,8].

Other factors that may impact ovarian function and disease include microplastics (MPs), nanoplastics (NPs), and trace elements. MPs are fragments of plastic with a particle size of ≤5 mm, whilst NPs have a particle size of ≤1 μm. Collectively, they are referred to as micro- and nanoplastics (MNPs). Being ubiquitous in the atmosphere and hydrosphere, they pose a potential health risk as they can accumulate in organs [9]. Trace elements, on the other hand, depending on the specific element, are essential components of biological structures: they play a role in the formation of the redox defence system and form part of enzymes that catalyse intracellular reactions. At the same time, they can be toxic at concentrations exceeding those required for their biological functions, or simply negatively impact cellular function [10].

This review aims to gather information on the impact of MNPs, as well as trace elements: Zinc (Zn), Selenium (Se), Copper (Cu), Chromium (Cr), Manganese (Mn), Cadmium (Cd), Lead (Pb), Arsenic (As) and Mercury (Hg), on the physiological function of the ovaries, as well as on conditions such as PCOS, ovarian dysfunction and infertility. We focus on both the individual effects of MNPs and trace elements, and their combined impact on ovarian cells (GCs and oocytes), describing their roles at the cellular and molecular levels. This convergent approach represents a unique contribution to the existing literature, as current studies focus mainly on analysing the effects of these molecules individually. The joint analysis of the effects of trace elements and MNPs sets this review apart from others already available and provides a comprehensive summary of the current state of knowledge and potential future research directions.

## 2. Materials and Methods

Methodologically, this article takes the form of a comprehensive narrative review. The literature was searched using the PubMed and Google Scholar databases. When developing the concept for the review, the literature was analysed, and trace elements with a potential impact on ovarian function, conditions such as PCOS, ovarian dysfunction and infertility, as well as elements interacting with MNPs, were identified. Elements essential for normal function (Zn, Se, Cu, Cr, Mn) and toxic elements (Cd, Pb, As, Hg) were taken into account. Literature relating to the effect of MNPs on the ovary was also selected for analysis.

The review analysed articles published between 1936 (the year of the first significant publication on this topic) and 2026, with the vast majority of the included literature having been published within the last 10 years to reflect the current state of knowledge. The following keywords and their combinations, using the Boolean operator ‘AND’, were employed in the search: ‘zinc’ OR ‘Zn’, ‘selenium’ OR ‘Se’, ‘copper’ OR ‘Cu’, ‘chromium’ OR ‘Cr’, ‘manganese’ OR ‘Mn’, ‘cadmium’ OR ‘Cd’, ‘arsenic’ OR ‘As’, ‘mercury’ OR ‘Hg’, ‘microplastics’ OR ‘MPs’, ‘nanoplastics’ OR ‘NPs’, ‘micronanoplastics’ OR ‘MNPs’ AND ‘ovary’, ‘polycystic ovary syndrome’ OR ‘PCOS’; ‘gonadal dysfunction’, ‘infertility’, ‘oxidative stress’, ‘cellular toxicity’, ‘trace elements’. Only peer-reviewed articles written in English were considered.

After identifying articles from the databases, duplicate records were removed using bibliographic management software (Mendeley, https://www.mendeley.com/) and manual screening. The remaining articles were screened on the basis of titles and abstracts in accordance with pre-defined selection criteria. Studies were included in the review that directly assessed the effect of trace elements and MNPs (measured on the basis of dietary intake, biomarkers in blood/urine or tissue concentrations) on ovarian function and pathology, as well as their interactions. The selected literature was subjected to a rigorous assessment of reliability and the risk of systematic error. Instead of using standard tools for the quantitative assessment of systematic error, a qualitative assessment was employed. Studies were evaluated using the following criteria: study design robustness, adequate sample size, the presence of appropriate control groups, and the consideration of confounding factors in clinical and epidemiological analyses. Emphasis was placed on original in vitro and in vivo experimental studies, prospective cohort studies and meta-analyses. The following were excluded from the review: non-peer-reviewed articles, case reports lacking broader mechanistic significance, biased literature reviews, and studies whose findings had been unequivocally refuted by subsequent high-quality studies.

Following the removal of duplicate articles, selection and critical quality assessment, a total of 167 articles were included in the review.

## 3. Characteristics and Effects of Microplastics in the Reproductive System

In this chapter, we discuss the sources of MNPs, the routes by which they enter the body, and their effect on ovarian function. We analyse the potential cellular mechanisms by which MNPs affect GCs, oocytes, and their function.

### 3.1. Sources of MNPs

The external environment is the source of MPs and NPs. These sources can be divided into primary and secondary. Primary MNPs are deliberately manufactured in the micron range and used in industry to produce cosmetics and personal care products. Secondary MPs, on the other hand, are formed through the fragmentation of macroplastics—for example, particles released from fabrics during washing due to mechanical friction. Both primary and secondary MNPs are present in terrestrial, atmospheric, and aquatic environments, where they circulate; rivers and lakes are estimated to be the main routes for the transfer of MNPs from terrestrial to aquatic environments. MPs suspended in the atmosphere also contribute to the MP cycle [11,12]. The effect of MNPs on human cells depends on several factors, including the type of polymer in the MNP molecule, the particle size and surface charge, and the MNP carrier [13].

### 3.2. Routes of Entry of MNPs into the Human Body

**Figure 1:**
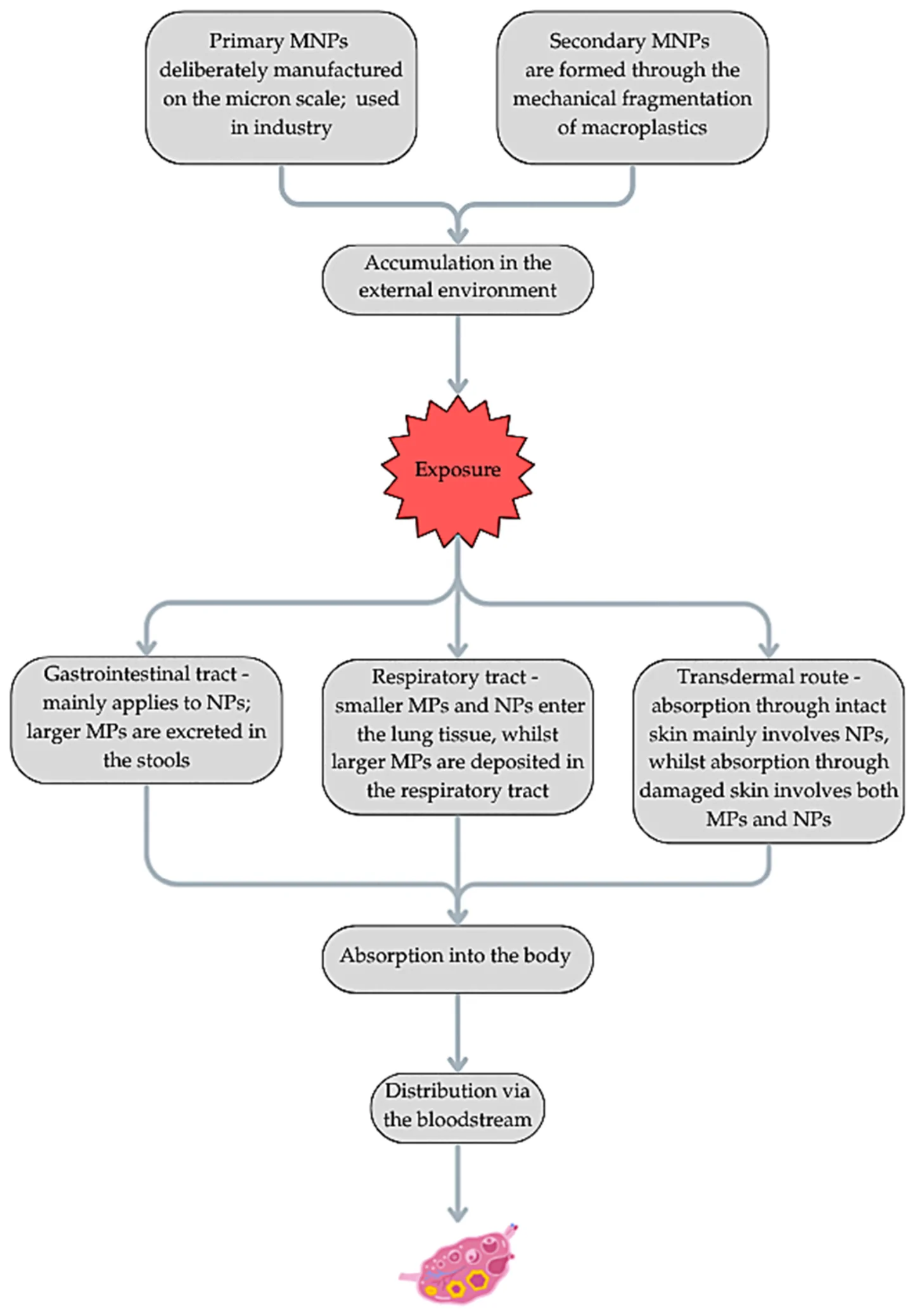
Sources and routes of MNPs entry. Abbreviations: MPs—microplastics; MNPs—micro- and nanoplastics; NPs—nanoplastics.

MPs and NPs can enter the human body via three routes: the digestive tract, the respiratory tract and the skin (Figure 1). As a result of consuming drinking water from plastic bottles, food stored in plastic containers and contaminated meat, MPs enter the human digestive tract. Although >90% of MPs are excreted in faeces after ingestion, smaller particles, in particular, may be absorbed through the intestinal epithelium, from which they are transported into the bloodstream [14]. Airborne MNPs, which constitute industrial air pollution, enter the respiratory tract, where they can penetrate to various depths: particles with a diameter of less than 2.5 µm can penetrate deep into lung tissue; those between 2.5 and 100 µm are considered inhalable and will settle in the smaller and larger airways, respectively [15]. In the context of transdermal absorption, there are four main routes of penetration: the transadnexal route, involving hair follicles and sweat glands, and the route through phospholipid membranes and the stratum corneum. The potential for transdermal penetration increases when the skin’s integrity is compromised by inflammatory processes or sunburn [16,17]. Regardless of the route by which they enter the body, MNPs can cross cellular barriers, enter the bloodstream, and thus reach various tissues and organs, such as the reproductive organs, liver, testicles, heart, kidney, spleen, intestine, the placenta, and the brain [18,19]. Further research should identify the other organs in which MNPs may accumulate, as the current findings are provisional.

### 3.3. The Effect of MNPs on Ovarian Function

The accumulation of MPs and NPs in the ovary damages oocytes, inhibits follicle growth, and causes chronic inflammation and fibrosis via collagen and fibronectin accumulation [20,21]. In granulosa cells (GCs), MPs induce oxidative stress (OS) and apoptosis through the Wingless-related integration site (Wnt)/β-catenin signalling pathway, and reduce anti-Müllerian hormone (AMH) levels [20,21]. Reactive oxygen species (ROS) overproduction also dysregulates the Hippo pathway, which controls primordial follicle activation, driving further GC apoptosis and stromal fibrosis [22].

MNPs directly disrupt mitochondrial function (a primary ROS source) and exacerbate OS by suppressing the Nuclear factor erythroid 2-related factor 2/Kelch-like ECH-associated protein 1 (Nrf2/Keap1) signalling pathway, thereby impairing antioxidant defences like superoxide dismutase (SOD) and glutathione (GSH) [23]. This redox imbalance activates the NLR family pyrin domain-containing 3 (NLRP3)/caspase-1 pathway, releasing interleukins (IL) IL-1β and IL-18, triggering granulocyte pyrolysis, and decreasing catalase (CAT), SOD, and GSH activity [24]. Similarly, activation of the Protein kinase RNA-like endoplasmic reticulum kinase—eukaryotic translation initiation factor 2 alpha—Activating Transcription Factor 4—C/EBP Homologous Protein (PERK-eIF2α-ATF4-CHOP) pathway impairs antioxidant enzymes, disrupts follicular development, and reduces oestrogen and progesterone production [25].

MNPs also induce ferroptosis by upregulating acyl-CoA synthetase (ACSL4), cyclooxygenase 2 (COX2), and ferritin heavy chain 1 (FTH1), while downregulating the member 11 of the solute carrier family 7 (SLC7A11) protein and glutathione peroxidase (GPX) type 4. Furthermore, MNPs disrupt the cytoskeletal architecture of GCs and oocytes by altering α-tubulin and dishevelled-associated activator of morphogenesis (DAAM-1) expression [26].

**Figure 2:**
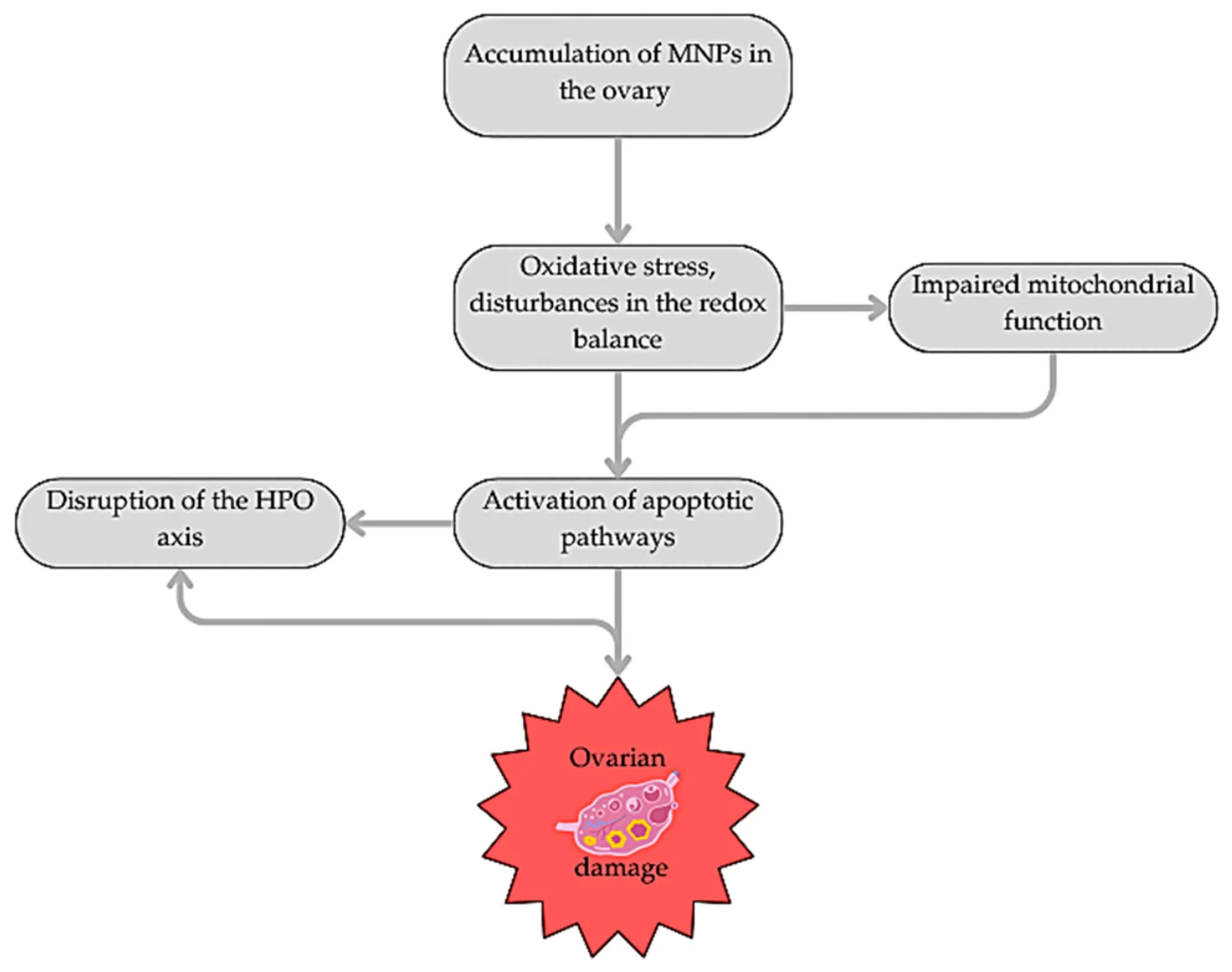
The effect of MNPs on the ovary. The arrows indicate the cause-and-effect relationship. The interaction between ‘Disruption of the HPO axis’ and ‘Ovarian damage’ is bidirectional, as damage to GCs and oocytes disrupts the functioning of the HPO axis, which in turn leads to further damage to ovarian cells. Importantly, the evidence base stems mainly from animal studies using high doses of MNPs, which limits the extent to which the findings can be extrapolated to humans. Abbreviations: HPO—hypothalamic–pituitary–ovary axis; MNPs—micro- and nanoplastics.

The changes described above may reduce ovarian reserve, potentially resulting in infertility [27] (Figure 2). Importantly, the evidence base stems mainly from animal studies using high doses of MNPs, which limits the extent to which the findings can be extrapolated to humans.

Damage to GCs and oocytes also disrupts the hormonal axis. As a result of damage to the ovarian cells via the mechanism described above, there is a reduction in the production of progesterone and oestrogen. This, in turn, may lead to increased HPG axis dysfunction, including elevated levels of LH and FSH, which consequently disrupt ovarian function [28].

Furthermore, studies in animal models and human organ samples suggest that MNPs may impair fertility through mechanisms other than simply affecting ovarian function. By accumulating in the uterine lining, they trigger inflammation and reduced receptivity, which significantly inhibit embryo implantation. Chronic inflammation can lead to fibrosis of the organ [29]. The accumulation of MNPs also affects males, where they can cause disruption to testicular cell function and spermatogenesis, resulting in reduced fertility [30].

## 4. The Effect of Trace Elements on Ovarian Function

**Figure 3:**
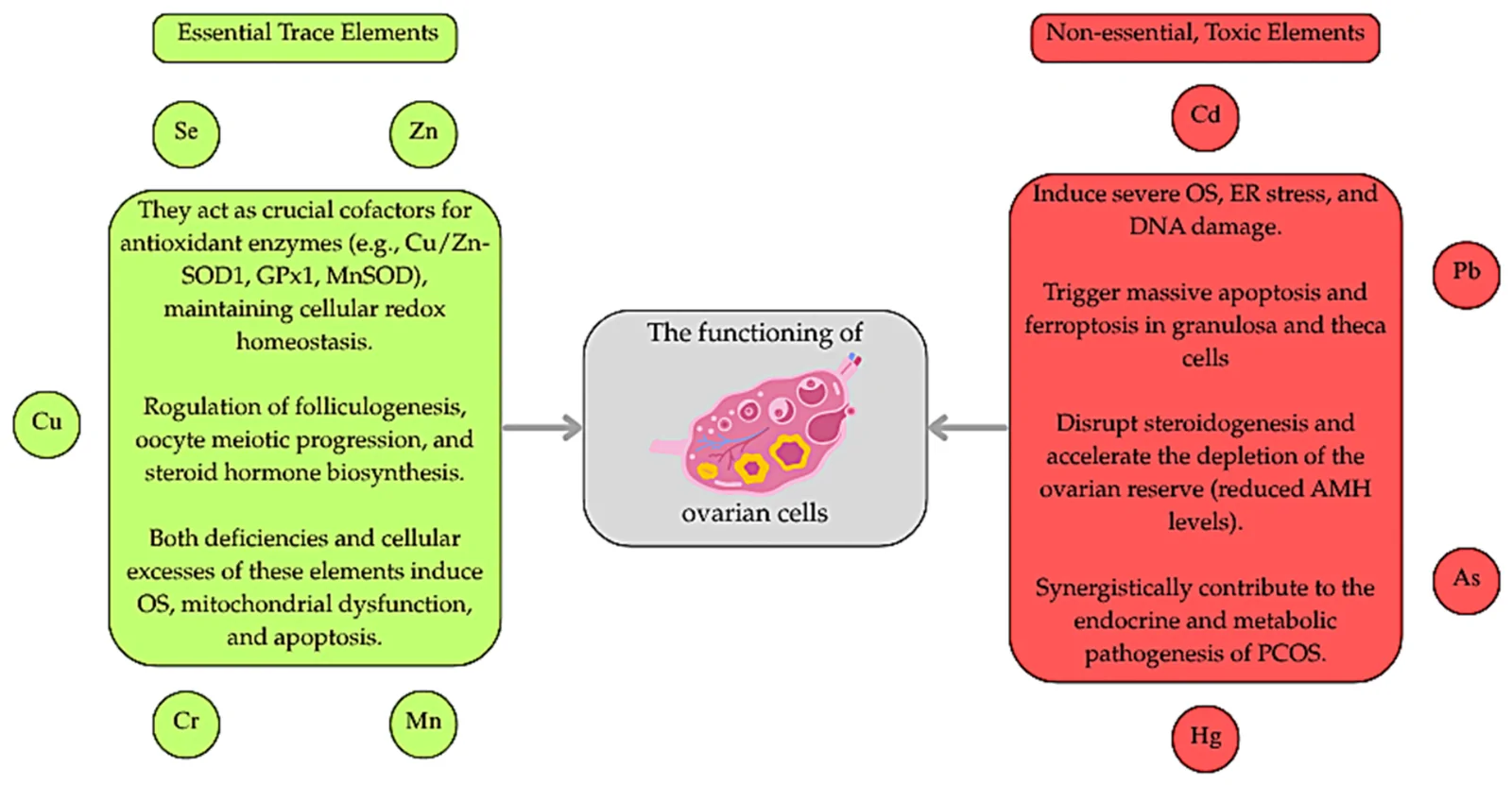
The effect of trace elements on ovarian function. Abbreviations: AMH—anti-Müllerian hormone; As—Arsenic; Cd—Cadmium; Cr—Chromium; Cu—Copper; Cu/ZnSOD1—Copper/Zinc superoxide dismutase type 1; ER—endoplasmic reticulum; GPx1—glutathione peroxidase type 1; Hg—Mercury; Mn—Manganese; MnSOD—manganese superoxide dismutase; OS—oxidative stress; Pb—Lead; PCOS—polycystic ovary syndrome; Se—Selenium; Zn—Zinc.

In this chapter, we describe the effects of the trace elements Cu, Zn, Se, Cr, Mn, Cd, Pb, As, and Hg on ovarian function (Figure 3). We describe their effects at the molecular and cellular levels, as well as at the level of the organ as a whole.

### 4.1. The Role of Essential Trace Elements in Ovarian Function

From a physiological perspective, essential bioelements such as Cu, Zn, Se, Cr, and Mn significantly influence ovarian homeostasis. Both deficiencies and excesses of extracellular and intracellular concentrations may result in cellular dysfunction.

#### 4.1.1. The Role of Zn

Zn is a crucial cofactor for numerous enzymes and transcription factors, including zinc-finger proteins regulating steroid hormone receptors for oestrogen, progesterone, and androgens. Intracellular homeostasis, governed by Zrt- and Irt-like protein (ZIP) and zinc transporter (ZnT) transporters along with metallothioneins, is vital for antioxidant defence (e.g., Cu/Zn-SOD1, GSH), inhibiting Nicotinamide adenine dinucleotide phosphate (NADPH) oxidase activity, and suppressing inflammatory signalling through Nuclear factor kappa B (NF-κB). Consequently, both Zn deficiency and excess disrupt redox balance, causing OS, mitochondrial dysfunction, and apoptosis [31,32].

Because ovarian folliculogenesis, steroidogenesis, and oocyte maturation require extensive cell proliferation and high metabolic activity, maintaining Zn homeostasis is essential for normal ovarian function. Zn participates in follicular development, granulosa cell differentiation, cumulus cell expansion, meiotic progression, ovulation, fertilisation, and early embryonic development. During folliculogenesis, ZIP and ZnT transporters regulate intracellular Zn levels, with stage-specific redistribution during follicle growth [33]. It promotes granulosa cell differentiation via the phosphorylated SMAD2/3 (pSMAD2/3) signalling pathway and reduces apoptosis [34], while accumulating during early follicle growth [35]. Zn is indispensable for meiotic progression to metaphase II; following fertilisation, the rapid release of intracellular Zn (“zinc sparks”) initiates early embryonic development [33].

Experimentally, Zn deficiency impairs ovarian physiology, causing morphologically abnormal oocytes and suppressed estrous cycling [36]. It also promotes mitochondrial dysfunction, DNA damage, and granulosa cell apoptosis, ultimately depleting the ovarian reserve and reducing AMH concentrations [37].

Clinical data on Zn status in women with PCOS are inconsistent, with studies reporting either reduced [38,39] or unchanged [40,41] serum Zn levels. Nevertheless, altered Zn status is strongly associated with insulin resistance, obesity, and lipid disturbances, suggesting it contributes directly to the metabolic manifestations of PCOS [42,43,44].

Overall, evidence shows that maintaining Zn balance is essential for maintaining ovarian physiology. Zn regulates folliculogenesis, granulosa cell survival, oocyte maturation, fertilisation, and early embryo growth by supporting antioxidant defences, mitochondrial health, hormonal signalling, and cell cycle regulation. Both Zn deficiency and Zn excess impair reproductive competence, although current evidence suggests that maintaining physiological Zn homeostasis is more important than the absolute Zn concentration. Despite substantial experimental evidence, further well-designed human studies are required to clarify the role of Zn in ovarian disorders, particularly PCOS and ovarian ageing.

#### 4.1.2. The Role of Se

Se exerts its essential biological functions primarily through selenoproteins, which maintain cellular redox homeostasis, support DNA and protein synthesis, and reduce heavy metal (As, Cd, Pb, Hg) toxicity [45,46,47,48]. In the ovary, precise redox regulation is vital; Glutathione peroxidase (GPX), particularly GPX1, plays a central role by regulating intracellular H_2_O_2_. Both deficiency and overexpression of GPX1 disrupt cellular homeostasis, leading to oxidative stress (OS) or reductive stress and insulin resistance, respectively [49].

Experimentally, Se promotes granulosa and luteal cell proliferation and 17β-estradiol (E2) biosynthesis by upregulating proliferation markers, including proliferating cell nuclear antigen (PCNA), cyclin-dependent kinase 1 (CDK1), phosphoinositide 3-kinase (PI3K), Akt, and adenosine monophosphate-activated protein kinase (AMPK) [47,50,51]. Furthermore, Se supplementation enhances antioxidant enzymes (GPX and SOD2) and key steroidogenic genes, including steroidogenic acute regulatory protein (StAR) and 3β-hydroxysteroid dehydrogenase (HSD) [47,50,51]. Conversely, Se deficiency induces ovarian degeneration and follicular atresia [47].

Clinically, women with PCOS consistently exhibit lower circulating Se, correlating with insulin resistance [52,53,54,55]. While Se supplementation may improve insulin sensitivity and reduce OS [56,57], Mendelian randomisation analyses suggest altered Se homeostasis is a consequence rather than a direct cause of PCOS [58].

Overall, Se is a vital trace element that supports ovarian health by forming selenoproteins that regulate redox homeostasis, inflammation, and cellular signalling. It aids in antioxidant defences, granulosa cell growth, follicle development, and hormone production, making it essential for normal ovarian function and female fertility. Se deficiency or abnormal selenoprotein activity can disrupt these processes, leading to changes in follicular dynamics and reduced ovarian function. While low Se levels are often seen in women with PCOS and linked to metabolic issues like insulin resistance, there is no conclusive evidence that Se deficiency directly causes the syndrome. More targeted research is needed to identify the ideal Se levels for ovarian health and explore its potential benefits in treating reproductive disorders.

#### 4.1.3. The Role of Cu

Cu acts as a crucial cofactor for enzymes like cytochrome c oxidase, Cu/Zn-SOD1, and ceruloplasmin [59]. Its homeostasis requires precise regulation via the epithelial Cu transporter 1 (CTR1), intracellular chaperones (Antioxidant 1 copper chaperone (ATOX1), Copper chaperone for superoxide dismutase (CCS), Cytochrome c oxidase copper chaperone 17 (COX17)), ATP7A/ATP7B transporters, and metallothioneins [60]. Disruptions in Cu balance compromise mitochondrial function, antioxidant defences, and cellular signalling [61].

Adequate Cu is essential for normal follicular development, luteal function, and ovulation [60,62,63], whereas elevated Cu concentrations correlate with follicular atresia [64] and HPO axis dysregulation via altered gonadotropin-releasing hormone (GnRH) and neurokinin B signalling [65].

Excessive Cu triggers OS via Fenton reactions, damaging DNA and cell membranes. This redox imbalance is associated with PCOS, leading to disrupted steroidogenesis and hyperandrogenism [61,66,67,68,69]. At the molecular and epigenetic levels, Cu overload disrupts local steroidogenesis in human granulosa cells by altering methylation patterns within the steroidogenic factor 1 (SF-1) gene promoter and modulating the FSHR/CYP19A1 pathway [70].

Cu homeostasis is essential for maintaining ovarian physiology. Both Cu deficiency and overload disrupt mitochondrial metabolism, antioxidant defence, inflammatory signalling, and cell survival pathways. Consequently, dysregulated Cu metabolism has been associated with impaired folliculogenesis, altered steroidogenesis, infertility, and ovarian disorders such as PCOS. Nevertheless, much of the available evidence derives from experimental animal models, highlighting the need for further human studies to clarify the role of Cu in ovarian physiology and pathology.

#### 4.1.4. The Role of Cr

Cr is a trace element long proposed to participate in carbohydrate and lipid metabolism by modulating insulin signalling pathways. Experimental studies indicate that trivalent Cr may enhance insulin receptor activity via the chromium-binding oligopeptide chromodulin, thereby improving insulin sensitivity and glucose utilisation [71]. However, despite extensive research over several decades, the physiological essentiality of Cr remains debated, as recent evidence has not convincingly demonstrated Cr deficiency in the general population, and its biochemical function has yet to be definitively established [72]. Nonetheless, pharmacological doses of Cr have been reported to produce beneficial metabolic effects in conditions characterised by impaired glucose homeostasis, making Cr particularly pertinent in disorders involving insulin resistance, including those affecting ovarian function [72].

Current evidence for a direct role of Cr in ovarian physiology is limited. Serum Cr levels are not significantly different between healthy women and those with PCOS [73]. Studies indicate that any benefits of Cr supplementation in PCOS are primarily related to improvements in systemic metabolic homeostasis rather than to direct modulation of ovarian function. Therefore, the role of Cr in folliculogenesis, steroidogenesis, and ovarian cell function remains unclear [55,57,74,75,76].

In contrast, hexavalent Cr [Cr(VI)] is a well-known reproductive toxicant that directly damages the ovary. Experimental studies have shown that Cr(VI) exposure disrupts follicular development, increases follicular atresia, and impairs steroidogenesis by inducing epigenetic alterations that suppress genes involved in ovarian development, cell proliferation, and survival, while activating apoptotic pathways [77].

In summary, current evidence does not indicate a significant direct physiological role for Cr in ovarian function. The benefits of Cr supplementation in reproductive issues mainly seem to result from improved systemic metabolic regulation rather than direct effects on GCs and oocytes. Conversely, exposure to Cr(VI) is definitively linked to ovarian toxicity, interfering with follicular development, hormone production, and cell viability. Additional research is needed to determine if trivalent Cr has any direct biological effects within the ovarian environment.

#### 4.1.5. The Role of Mn

Mn is an essential trace element and a critical cofactor for MnSOD, which maintains cellular redox homeostasis and protects mitochondria from oxidative damage [78,79]. In the ovary, MnSOD is expressed in GCs, theca interna cells, and luteal cells, where it neutralises mitochondrial superoxide radicals generated during steroidogenesis [80,81]. Mn indirectly supports steroid biosynthesis through the close relationship between MnSOD and Ad4-binding protein, a vital regulator of steroidogenic enzyme expression. Furthermore, FSH-stimulated follicular growth correlates with increased MnSOD expression, mitigating granulosa cell apoptosis and atresia [81]. Mn preferentially accumulates in mitochondria-rich structures, such as Graafian follicles and the corpus luteum, particularly during peak progesterone production, ovulation, and luteal development. Consequently, Mn deficiency leads to reduced fertility, follicular abnormalities, and cystic ovarian changes [78,82].

Conversely, excessive Mn exposure is toxic [78,83]. In vitro studies reveal dose- and time-dependent cytotoxicity characterised by genomic instability, micronuclei formation, and diminished cell viability [84]. In mouse models, Mn overload increases ovarian OS and inflammatory cytokine production, while concurrently disrupting steroidogenic enzyme activity and hormonal homeostasis [85].

Clinically, while some studies report lower serum Mn levels in women with PCOS —potentially reflecting increased utilisation by antioxidant systems like MnSOD [86]—recent meta-analyses have found no significant differences compared to healthy controls, leaving current evidence inconclusive [73].

In summary, Mn is an essential trace element that plays a pivotal role in maintaining ovarian homeostasis. Its primary function is serving as a cofactor of MnSOD, which is essential for preserving mitochondrial function, redox balance, and steroidogenic processes. Adequate Mn levels are critical for supporting follicular development, luteal functionality, and granulosa cell viability. Conversely, both deficiencies and excesses of Mn can detrimentally affect ovarian physiology by inducing OS, impairing mitochondrial function, and triggering apoptosis. While disruptions in Mn homeostasis have been linked to reproductive disorders, including PCOS, current clinical evidence does not suffice to establish a direct causal relationship definitively.

### 4.2. The Role of Toxic Trace Elements in Ovarian Function

Unlike essential elements, toxic elements have a detrimental effect on ovarian cells at any concentration. These elements include: Cd, Pb, As and Hg. These metals may affect the granulosa cells of the ovary by causing OS both directly and indirectly, damaging them, disrupting the HPG axis and having a real impact on women’s reproductive health [87].

#### 4.2.1. The Role of Cd

Cd is a heavy metal that impairs ovarian function. Its main sources are industrial pollution and agricultural fertilisers. Due to its long half-life and slow rate of excretion, which lead to its bioaccumulation in the body, cadmium is particularly dangerous. Its accumulation in the ovaries may interfere with the processes of folliculogenesis and oogenesis; numerous studies point to its direct toxicity towards both GCs and theca cells, which provide vital nutritional and hormonal support for the maturing oocyte [88]. Cd induces severe OS and mitochondrial damage, triggering excessive mitophagy and apoptosis, which reduces oocyte quality and causes premature ovarian failure [89]. Additionally, Cd-induced autophagy releases intracellular Fe^2+^, driving the ferroptotic destruction of cell membranes [90,91].

In human GCs, Cd causes dose-dependent structural damage (loss of adhesion, shrinkage) and disrupts intercellular communication, directly inhibiting progesterone synthesis [92]. In theca cells, Cd-induced mitochondrial bioenergetic collapse impairs androgen synthesis, depriving GCs of oestrogen substrates and disrupting follicular hormonal balance [93]. Early folliculogenesis is also targeted; Cd dysregulates stem cell factor and its receptor (SCF/c-kit) gene expression, depleting the primary ovarian reserve [94]. Ultrastructurally, Cd damages the cumulus–oocyte complex by causing mitochondrial cristae degradation, ER expansion, and chromatin condensation [95,96].

Genetically and epigenetically, Cd reduces mitosis and meiosis phases and dysregulates the ceRNA network and transcriptome, shortening fertility [97]. Strikingly, paternal Cd exposure induces epigenetic changes in sperm that impair GC hormone synthesis in unexposed female offspring [98]. Co-exposure with other metals (e.g., Hg) causes severe ovarian structural damage [99]. Clinically, blood Cd negatively correlates with AMH, indicating accelerated follicular reserve depletion [100].

Cd also plays a significant role in the pathogenesis of PCOS. Studies based on the analysis of follicular fluid confirm that the direct accumulation of Cd in the follicular microenvironment dysregulates local steroidogenesis, and directly disrupts the central HPO axis. This clinically manifests as an abnormal LH/FSH ratio, a key mechanism mediating the development of the PCOS phenotype [101]. Systemically, elevated Cd is associated with increased total testosterone and AMH, reflecting inhibited follicle maturation [102], and its synergy with other heavy metals further increases the risk of developing clinical PCOS [103].

To summarise, Cd causes multi-pathway damage to the ovaries, destroying GCs and theca by inducing apoptosis, ferroptosis and severe OS. As a result, oocyte maturation is inhibited, sex hormone synthesis is disrupted, and ovarian reserve is depleted. Studies link exposure to this metal with a decrease in AMH levels, a higher risk of PCOS and the potential for epigenetic transmission of these disorders to subsequent generations.

#### 4.2.2. The Role of Pb

Pb structurally damages the ovary, causing stroma oedema, vascular degeneration (microthrombosis), and follicular atresia [104]. A key aspect of lead’s toxic effects is the disruption of the HPO axis at the receptor level. Studies on granulosa cells have shown that lead acetate reduces the ability of pituitary hormones (LH and FSH) to bind to their surface receptors, which directly disrupts the physiological feedback loop of the axis and impairs 17β-HSD activity [105]. Clinically, elevated blood Pb in in vitro fertilisation (IVF) patients activates the unfolded protein response (UPR) and ER stress, leading to GCs apoptosis and reduced oocyte quality [106].

During puberty, Pb exposure disrupts folliculogenesis and steroidogenesis via the activation of ER stress and the IRE1α-JNK signalling pathway, which induces massive GC apoptosis and reduces sex hormone levels [107]. Both acute and chronic Pb exposures cause significant Pb accumulation in ovarian tissue, triggering massive atresia of antral follicles and depleting the primary follicle pool [108]. Furthermore, lactational exposure to Pb impairs the prepubertal ovarian reserve, though these morphological impairments are partially reversible following the cessation of exposure [109].

Pb exposure is positively associated with an increased risk of PCOS. Pb acts alone or synergistically with other metals to disrupt the sex hormone profile [103]. In women with PCOS, higher blood Pb concentrations correlate with peripheral OS and chronic low-grade inflammation, driving insulin resistance and hyperandrogenism [110].

Pb causes multi-level damage to ovarian tissue, disrupting folliculogenesis and accelerating follicular atresia. The stress on the ER caused by this heavy metal, together with reduced activity of key enzymes, impairs GC function and the synthesis of sex hormones. Furthermore, the presence of Pb in the body promotes OS and inflammation, which directly exacerbates the metabolic and hormonal disorders underlying PCOS.

#### 4.2.3. The Role of As

As induces severe redox imbalance in the exceptionally sensitive ovarian tissue, generating ROS, increasing MDA, and depleting antioxidant enzymes: SOD, GSH, and CAT. This directly stimulates follicular atresia prior to visible hepatic or renal toxicity [111,112].

Molecularly, As disrupts DNA repair in GCs by inducing the proteasomal degradation of RNA helicase DDX5. This impairs FANCA gene alternative splicing, leading to R-loop accumulation, DNA double-strand breaks (γ-H2AX foci), and premature follicular ageing [113]. Chronic As exposure also promotes an inflammatory phenotype in ovarian surface epithelial cells, marked by IL-6 and IL-8 release [114]. During puberty, As reduces the ovarian reserve and fertility by blocking the PKA-ERK-JNK-cJUN signalling pathway, drastically reducing oestrogen, progesterone, and testosterone levels [115].

Furthermore, As impairs steroidogenesis through epigenetic modifications; it induces hypermethylation of the SF-1 gene promoter, silencing its expression and downregulating key steroidogenic proteins (StAR, CYP11A1, and CYP19A1), thereby drastically decreasing E2 levels [116].

Epidemiologically, elevated peripheral As concentrations are strongly associated with PCOS risk [103,117]. In PCOS patients, As accumulation correlates with central HPO axis dysregulation (abnormal LH/FSH ratio), insulin resistance, inflammation, and OS [103,118].

Exposure to As induces severe OS, inflammation and DNA damage in the ovaries, leading to massive follicular atresia and a reduction in ovarian reserve, as this tissue is more sensitive than other organs. This compound also disrupts steroidogenesis by silencing key proteins and enzymes (including StAR and CYP11A1), which drastically reduces oestrogen and progesterone levels, contributing to infertility and the development of PCOS.

#### 4.2.4. The Role of Hg

Hg induces multifaceted ovarian toxicity. In vivo studies reveal that chronic exposure to Hg (e.g., HgCl_2_ or Hg vapours) prolongs the inter-oestrus phase, decreases serum progesterone and E2 concentrations, and structurally damages the corpus luteum [119,120]. Furthermore, Hg significantly reduces the total number of primary, growing, and Graafian follicles while increasing follicular atresia, tissue oedema, and hydropic degeneration of GCs [121]. At the cellular and genetic levels, Hg induces dose- and time-dependent DNA strand breaks [122], severe OS (evidenced by increased MDA levels) [123], and apoptosis in GCs, which directly inhibits progesterone secretion [124].

Beyond direct cytotoxicity, Hg is a significant predisposing factor for the PCOS phenotype and its associated metabolic abnormalities. Chronic Hg exposure induces ovarian cysts, pathological fat droplet accumulation, OS, and systemic metabolic disorders, including insulin resistance and glucose intolerance [125]. Clinically, women with PCOS exhibit significantly higher serum and follicular fluid Hg concentrations, which correlate directly with a decreased antioxidant reserve (reduced GSH levels) and metabolite disturbances within the oocyte microenvironment [101,118]. Additionally, population-based studies demonstrate that increasing Hg exposure accelerates the decline in AMH levels, directly contributing to the premature depletion of the follicular reserve [126].

Exposure to Hg causes multifaceted toxicity in the ovaries by inducing OS, DNA damage and disturbances in steroidogenesis and gonadotropin sensitivity. These phenomena lead directly to the development of changes characteristic of the PCOS phenotype, a reduction in oocyte quality and accelerated depletion of the ovarian reserve.

## 5. Molecular Convergence: Pathogenic Mechanisms and Toxic Synergism Between Trace Elements and MNPs in the Ovary

### 5.1. Mechanistic Convergence of Zn and MNPs Toxicity in the Ovary

The available evidence suggests that Zn and MPs may converge on several molecular and cellular processes involved in ovarian dysfunction. However, direct experimental evidence concerning their combined effects in ovarian tissue remains limited. The principal mechanisms potentially shared by Zn-related toxicity and MP-induced ovarian injury include disruption of redox homeostasis, mitochondrial dysfunction, altered autophagy and apoptosis, impairment of follicular development, and endocrine dysregulation. These mechanisms may be particularly relevant when Zn homeostasis is disrupted, as both Zn deficiency and excessive exposure to Zn-based nanoparticles may adversely affect GCs and oocytes through distinct yet partially overlapping pathways [20,37,127].

Disruption of redox homeostasis represents a major potential point of convergence. In ovarian tissue, Zn deficiency has been associated with mitochondrial dysfunction and increased OS. In contrast, exposure to zinc oxide nanoparticles (ZnO-NPs) can generate ROS and induce OS in developing oocytes. Similarly, MP exposure has been shown to increase ROS production, promote lipid peroxidation, impair antioxidant defences, and induce oxidative damage in ovarian tissue and GCs. Thus, both altered Zn homeostasis and MP exposure may converge on excessive ROS generation and impaired antioxidant capacity, potentially leading to oxidative damage in GCs and oocytes [20,37,127].

Mitochondrial dysfunction may represent a second important point of molecular convergence. Zn deficiency has been shown to disrupt mitochondrial dynamics and function in ovarian tissue, resulting in OS. This can impair follicular development and reduce oocyte quality [37]. MP exposure has likewise been associated with mitochondrial dysfunction, altered energy metabolism, depletion of intracellular glutathione, and increased OS in mammalian oocytes [128]. These findings suggest that disruption of mitochondrial homeostasis may provide a common mechanistic link between altered Zn status and MPs-induced ovarian toxicity. Mitochondrial impairment may further amplify ROS generation and promote downstream activation of cell-death pathways [37,128].

Apoptosis and dysregulated cellular quality-control mechanisms constitute another potential area of convergence. Exposure to ZnO-NPs has been shown to activate autophagy and apoptosis in developing oocytes and to induce additional forms of cell death, including necroptosis, accompanied by OS and impaired follicular development [127]. MPs have similarly been associated with apoptosis of GCs, as well as impaired follicular development and oocyte quality [20,22]. In addition, Zn deficiency may impair autophagy in ovarian tissue and increase apoptosis, suggesting that disturbances in the balance among cellular survival, autophagy, and programmed cell death may contribute to ovarian dysfunction [37].

The convergence of these mechanisms may ultimately impair folliculogenesis and oocyte competence. Zn deficiency has been associated with reductions in the numbers of total and mature follicles, decreased anti-Müllerian hormone levels, impaired oocyte maturation, and reduced oocyte quality [37]. MP exposure has likewise been linked to follicular atresia, reduced follicular development, impaired oocyte maturation, and decreased ovarian reserve-related functions [20,28]. Therefore, disturbances in Zn homeostasis and MP-induced cellular injury may converge functionally to impair follicular growth and maturation [20,28,37].

Endocrine and steroidogenic disruption may represent an additional shared outcome. Zn is an essential regulator of reproductive physiology, and altered Zn status may affect hormone secretion and follicular maturation. Exposure to ZnO-NPs has also been shown to affect ovarian follicular steroidogenic function and E2 production [129]. MP exposure has similarly been associated with altered sex hormone levels, disruption of ovarian endocrine function, and impaired steroidogenic processes [28]. These findings suggest that both altered Zn homeostasis and MP exposure may interfere with ovarian endocrine function. However, the direction and magnitude of these effects may depend on the chemical form, dose, and duration of Zn exposure [28,129].

Overall, the available evidence supports a mechanistic convergence model in which dysregulated Zn homeostasis and MP exposure may affect the ovary through interconnected pathways involving OS, mitochondrial dysfunction, impaired autophagy, apoptosis, follicular injury, and endocrine disruption. A proposed sequence is: altered Zn homeostasis and/or MP exposure → redox imbalance and increased ROS production → mitochondrial dysfunction → impaired cellular quality control and activation of apoptosis → follicular and oocyte injury → impaired steroidogenesis and ovarian function. However, because direct studies of combined Zn–MP exposure in the ovary are currently scarce, these mechanisms should be interpreted as biologically plausible points of convergence rather than definitive evidence of synergistic toxicity. Future studies should specifically investigate combined exposure, Zn speciation and dose, particle characteristics, ovarian accumulation, and formal dose–response interactions to determine whether the combined effects are additive, antagonistic, or synergistic.

### 5.2. Mechanistic Convergence of Se and MNPs Toxicity in the Ovary

Within the literature identified for this review, no experimental study has directly evaluated the combined effects of Se and MPs on ovarian tissue. Consequently, their potential interaction must currently be considered indirectly, using evidence from studies examining each factor separately in ovarian models and from co-exposure studies conducted in other biological systems. Such comparisons may indicate mechanistic plausibility but cannot demonstrate that Se modifies MPs-induced ovarian toxicity in vivo.

Unlike MPs, Se is an essential micronutrient whose biological effects depend on its chemical form and dose, with both deficiency and excessive intake potentially being harmful. At appropriate levels, Se contributes to ovarian redox homeostasis through selenoproteins, particularly GPX1, GPX3, and GPX4 [47,130].

In ageing mice, Se deficiency was associated with reduced antioxidant capacity, increased ovarian apoptosis, fewer primordial, primary, and growing follicles, and impaired oocyte developmental competence. Conversely, supplementation with either inorganic or organic Se improved follicle maintenance, reduced ovarian apoptosis, increased blastocyst development, and enhanced ovarian expression of *Gpx1*, *Gpx3*, *Gpx4*, and *Selenof* relative to Se-deficient animals [130].

The human evidence is more limited and less conclusive. A meta-analysis of clinical trials in women with PCOS showed that Se supplementation increased total antioxidant capacity but did not significantly improve testosterone, insulin resistance, glutathione, malondialdehyde, lipid parameters, or body weight. The authors therefore did not support routine Se supplementation, although a potential benefit was suggested for selected patients with OS-related impairment of follicle quality [131].

In contrast, exposure to ovarian MPs has predominantly been associated with disruption of redox homeostasis. In rats, 0.5 µm polystyrene MPs (PS-MPs) entered GCs, reduced the number of growing follicles and serum AMH, and induced granulosa-cell apoptosis and ovarian fibrosis. These effects were linked to OS-dependent activation of Wnt/β-catenin signalling, as antioxidant treatment attenuated both granulosa-cell apoptosis and pathway activation [20].

Thus, OS represents the clearest potential point of convergence between Se and MP effects in the ovary. MPs promote oxidative injury, apoptosis, fibrosis, and loss of ovarian reserve, whereas adequate Se status may support selenoprotein-mediated antioxidant protection and follicular survival. Nevertheless, this apparent mechanistic opposition does not establish that Se supplementation can prevent MPs-induced ovotoxicity, particularly given the narrow optimal range and U-shaped biological response to Se [132].

Direct co-exposure studies in mouse liver consistently indicate that nano-Se attenuates MPs-induced hepatotoxicity through overlapping antioxidant, anti-inflammatory, mitochondrial, and metabolic mechanisms. Se nanoparticles reduced ROS generation, lipid peroxidation, hepatic lipid accumulation, serum liver enzyme elevations, and histopathological injury, while restoring GSH, SOD, and CAT activity [133,134]. Comparable protection was observed in the liver and intestine of grass carp. Dietary nano-Se reduced MPs-induced histological injury, moderated disturbances in antioxidant biomarkers, and lowered the expression of genes associated with immune activation and inflammation [132]. However, given the lack of direct evidence concerning the ovaries, this conclusion is speculative and requires further research.

Taken together with the separate ovarian studies, these findings support the biological plausibility that appropriate Se availability or nano-Se supplementation may mitigate selected effects of MP exposure. MPs impair ovarian function predominantly through OS-driven granulosa-cell apoptosis, fibrosis, and follicular loss, whereas appropriate Se availability supports selenoprotein-dependent antioxidant defence, follicular survival, and oocyte competence. The non-ovarian co-exposure studies directly demonstrate that nano-Se can counteract several corresponding effects of MPs, including OS, lipid peroxidation, inflammation, mitochondrial dysfunction, and tissue injury. Nevertheless, this protection has been demonstrated in non-ovarian models and cannot yet be extrapolated to ovarian tissue, conventional Se compounds, or varying Se doses.

### 5.3. Mechanistic Convergence of Cu and MNPs Toxicity in the Ovary

A comprehensive analysis of the available studies indicates that Cu and MPs exhibit significant molecular convergence: despite their different chemical natures, they activate similar pathogenic pathways that lead to impaired ovarian function. The best-understood common mechanism of action for both factors is the induction of oxidative stress. In both cases, there is a reduction in antioxidant enzyme activity, an increase in lipid peroxidation, and an accumulation of oxidative damage in GCs and oocytes. A disruption of the oxidative balance serves as the starting point for further changes [25,81,135].

A common mechanism of action for Cu and MPs is mitochondrial dysfunction. In both cases, there is an imbalance between the cell’s energy requirements and the mitochondria’s ability to meet them, which particularly affects GCs and oocytes that require high metabolic activity during folliculogenesis [25,136,137].

Molecular convergence also involves the inhibition of proliferation and impaired survival of granulosa cells. Excessive exposure to Cu may inhibit the proliferation of these cells; MPs exhibit a similar effect. Consequently, both factors lead to reduced granulosa cell viability, increased susceptibility to apoptosis, and disruption of the normal environment necessary for the maturation of ovarian follicles [20,22,138].

Another key aspect of their combined action is the disruption of steroidogenesis. The combined effect of these factors is a disruption of GCs’ endocrine function and abnormal communication between the oocyte and the follicular supporting cells, which may lead to impaired ovarian follicular maturation [70,139,140].

Both groups of pollutants also exhibit genotoxic potential, primarily due to increased OS. The combined effect of these two factors is increased genomic instability and reduced ability of GCs to regenerate properly and maintain homeostasis [25,135].

Although there are still few direct studies on co-exposure to Cu and MPs in the ovary, the similarity of the observed molecular mechanisms suggests a potential toxic synergy between the two factors. Both Cu and MPs activate common pathways involving OS, mitochondrial dysfunction, impaired steroidogenesis, inhibition of granulosa cell proliferation and impaired ovarian follicle development. This suggests that concurrent exposure to Cu and MPs may constitute a significant risk factor for normal reproductive function and justifies the need for further experimental and epidemiological studies [25,137,141,142].

### 5.4. Mechanistic Convergence of Cr and MNPs Toxicity in the Ovary

The available evidence indicates that Cr, particularly Cr(VI), and MPs may converge on several molecular and cellular mechanisms involved in ovarian toxicity. Although direct experimental evidence concerning combined Cr–MP exposure in ovarian tissue remains limited, studies investigating the individual effects of Cr(VI) and MPs identify several potentially shared pathways, including OS, mitochondrial dysfunction, DNA damage, apoptosis, follicular atresia, disruption of steroidogenesis, and impairment of ovarian function. These mechanisms may provide a biological basis for potential interactions during combined exposure, although the nature of such interactions requires direct experimental confirmation [20,25,143,144].

OS represents one of the most important potential points of convergence between chromium- and MP-induced ovarian toxicity. The intracellular reduction of Cr(VI) generates reactive intermediates and ROS, which can lead to oxidative damage and deplete antioxidant defences. In the ovary, Cr(VI) exposure has been associated with increased OS and reduced antioxidant enzyme activity, including alterations in GPX, CAT, thioredoxin, and peroxiredoxin systems. Pharmacological attenuation of OS reduced Cr(VI)-induced ovarian injury, supporting the role of redox imbalance in chromium-induced ovotoxicity [143,144]. Similarly, MP exposure has been associated with increased ROS production, lipid peroxidation, impaired antioxidant defences, and oxidative damage in ovarian tissue and GCs [20,25]. Thus, both Cr(VI) and MPs may converge on excessive ROS generation and impaired antioxidant capacity, potentially increasing oxidative damage in GCs and oocytes [20,25,143,144].

Mitochondrial dysfunction represents another potential shared mechanism. Chromium-induced ovarian toxicity has been associated with mitochondrial involvement in apoptosis, including cytochrome c release and activation of downstream apoptotic pathways in GCs. Cr(VI) exposure has also been shown to induce mitochondrial dysfunction in oocytes and to impair oocyte competence through oxidative damage and disruption of cellular structures involved in meiotic maturation [145,146]. MNPs similarly affect mitochondrial function, including disruption of mitochondrial membrane potential, abnormal mitochondrial morphology, impaired energy metabolism, and increased OS in GCs and oocytes [23,147]. Therefore, mitochondrial dysfunction may be a common mechanistic link by which Cr and MPs amplify ROS generation and activate cell death pathways [23,145,146,147].

DNA damage represents an additional point of mechanistic convergence. Cr(VI) is capable of inducing oxidative DNA damage and DNA double-strand breaks in oocytes, accompanied by activation of DNA repair responses and abnormalities in meiotic chromosome segregation [146]. Cr exposure has also been associated with epigenetic alterations in the ovary, including changes in DNA methylation and histone modifications that affect genes involved in ovarian development, cell survival, and apoptosis [77]. MP exposure can likewise induce OS-associated cellular injury and genomic damage, particularly through excessive ROS production and mitochondrial dysfunction. Consequently, the combined effects of Cr and MPs may potentially converge on oxidative DNA damage and impaired genomic stability. However, direct evidence for this mechanism during combined exposure in ovarian tissue remains unavailable [77,146].

Apoptosis constitutes a major downstream consequence of these convergent mechanisms. Cr(VI) exposure induces apoptosis in ovarian GCs and germ cells through activation of mitochondrial and p53-associated pathways, including cytochrome c release, increased expression of pro-apoptotic proteins, activation of caspase-3, and suppression of anti-apoptotic factors [145,148]. Cr(VI)-induced epigenetic changes have also been linked to repression of genes involved in cell survival and activation of apoptotic pathways, ultimately contributing to follicular atresia [77]. MPs similarly induce apoptosis in GCs and ovarian tissue through OS and associated signalling pathways, including activation of apoptotic signalling and impairment of cell-survival pathways [20,22,147]. Accordingly, OS, mitochondrial dysfunction, and DNA damage may represent convergent upstream mechanisms leading to ovarian cell apoptosis following exposure to either toxicant [20,22,77,145,147,148].

The convergence of these cellular mechanisms may ultimately result in follicular atresia and impaired folliculogenesis. Cr(VI) exposure has been associated with increased follicular atresia, disruption of follicular development, and reduced ovarian function [77,143]. MP exposure has likewise been associated with increased follicular atresia, reduced numbers of growing follicles, granulosa-cell apoptosis, and impairment of ovarian reserve-related functions [20,25,147]. Therefore, combined oxidative, mitochondrial, genomic, and apoptotic injury may converge functionally to result in the loss of healthy follicles and disruption of ovarian tissue homeostasis [20,25,77,143,147].

Endocrine disruption and impaired steroidogenesis represent additional shared functional outcomes. Cr(VI) exposure has been shown to disrupt ovarian steroid hormone biosynthesis and oestrogen metabolism, with decreased E2 production and altered expression of factors involved in steroidogenesis [143,144]. MP exposure has similarly been associated with altered sex hormone levels, impaired ovarian endocrine function, and disruption of steroidogenic processes [25,28]. These findings suggest that Cr and MPs may converge on ovarian endocrine dysfunction through interconnected effects on follicular integrity, granulosa-cell function, OS, and steroidogenic pathways [25,28,143,144].

Overall, the available evidence supports a mechanistic convergence model in which Cr(VI) and MPs may affect the ovary through interconnected oxidative, mitochondrial, genomic, apoptotic, and endocrine pathways. A proposed sequence is: Cr(VI) and/or MP exposure → increased ROS production and redox imbalance → mitochondrial dysfunction and oxidative DNA damage → activation of apoptotic and stress-response pathways → granulosa-cell and oocyte injury → follicular atresia and impaired steroidogenesis → ovarian dysfunction. However, because direct studies of combined chromium–MP exposure in the ovary are currently scarce, these mechanisms should be interpreted as biologically plausible points of convergence rather than definitive evidence of synergistic toxicity. Future studies should directly investigate combined exposure, Cr speciation [particularly Cr(III) versus Cr(VI)], particle characteristics, ovarian accumulation, oxidative and mitochondrial endpoints, DNA damage, and formal dose–response interactions to determine whether the combined effects are additive, antagonistic, or synergistic.

### 5.5. Mechanistic Convergence of Mn and MNPs Toxicity in the Ovary

Among the literature identified for this review, no study has directly examined the combined effects of Mn and plastic particles on ovarian tissue. Their potential interaction must therefore be considered indirectly by comparing studies of each contaminant separately in ovarian models and by examining available co-exposure studies conducted in non-ovarian models. Such evidence may establish mechanistic plausibility but cannot demonstrate that the same interaction occurs in the ovary.

Mn is an essential trace element, but excessive exposure can disrupt female reproductive function. In animal models, Mn accumulated in ovarian tissue and altered the development and function of reproductive organs. Exposure reduced ovarian weight in immature rats and disrupted gonadotropin secretion [149], while a separate model demonstrated ovarian OS, inflammation, dysfunction of steroidogenic enzymes, and hormonal imbalance [85].

Both Mn and MPs induce marked, model-dependent endocrine disruption and alter sex hormone dynamics. In animal models, excessive Mn accumulates in ovarian tissue, reduces ovarian weight, and alters gonadotropin secretion [149]. Hormonal responses to Mn exhibit complex dose- and model-dependent patterns: Mn exposure reduced FSH in immature rats and adult mice, whereas LH declined dose-dependently, despite central Mn administration acutely stimulating GnRH-dependent LH release [85,149,150]. Furthermore, Mn directly impairs local ovarian steroidogenesis, decreasing progesterone, increasing E2, and altering the enzymatic activities of 3β- HSD1, 11β-HSD1, and 17β-HSD1 [85]. Exposure to MPs produces a similarly marked but variable endocrine phenotype, ranging from elevated serum FSH, E2, and testosterone in rats to reduced E2, LH, and FSH accompanied by altered steroidogenic gene expression in zebrafish [151,152]. Thus, both contaminants converge on disrupting HPO axis feedback and localised steroid biosynthesis, although the direction of hormonal shifts depends on exposure duration, dose, species, and particle characteristics [85,149,150,151,152].

Oxidative and cellular injury represent additional central components of shared toxicity. Mn exposure induces lipid peroxidation, depletes intracellular glutathione, elevates pro-inflammatory cytokines (IL-8, IL-12, IL-15, IL-18), and exerts concentration- and time-dependent cytotoxic and genotoxic effects in ovarian-derived cells [84,85]. PS-MPs induce a partly overlapping ovarian phenotype, characterised by ROS-dependent granulosa-cell apoptosis, reduced AMH levels, depletion of growing follicles, and transforming growth factor beta (TGF-β)-mediated ovarian fibrosis [20]. While MP-induced ovotoxicity primarily centres on ROS-driven granulosa-cell apoptosis and fibrotic remodelling, Mn exerts broader inflammatory, neuroendocrine, and genotoxic effects. Nevertheless, OS serves as a central upstream mechanism through which both exposures impair granulosa-cell survival and follicular maintenance [20,84,85].

Direct evidence from non-ovarian reproductive models demonstrates that Mn–plastic interactions are highly dependent on particle dimension. Submicron NPs (0.1 µm) exhibit a significantly higher sorption capacity for Mn and enter cells more efficiently than larger particles (1–5 µm). Crucially, higher Mn adsorption onto NPs does not translate into a measurable increase in total tissue Mn accumulation; rather, co-exposure modifies Mn absorption, membrane interactions, and intracellular distribution, amplifying biological toxicity through post-transcriptional and cell-cycle mechanisms. Although demonstrated in male reproductive models, these findings highlight the biological plausibility that small NPs may physically alter Mn bioavailability and intensify its cellular toxicity without necessarily increasing total tissue burden [153].

Overall, the available evidence supports a mechanistic convergence model in which Mn and MPs act on the ovary through interconnected neuroendocrine, oxidative, inflammatory, and steroidogenic pathways. A proposed sequence is: excessive Mn and/or MP exposure → particle-size-dependent cellular uptake and sorption kinetics → redox imbalance, increased ROS generation, and pro-inflammatory cytokine release → disruption of central HPO axis regulation and localised steroidogenic enzyme dysfunction → granulosa-cell apoptosis, oxidative injury, and impaired follicular maturation → follicular atresia and ovarian endocrine dysfunction. Because direct co-exposure studies in mammalian ovaries remain lacking, these mechanisms represent biologically plausible points of convergence rather than definitive proof of toxicological synergy. Future research should specifically investigate combined exposure across distinct particle dimensions, Mn oxidation states, ovarian accumulation patterns, and formal dose–response interaction models.

### 5.6. Mechanistic Convergence of Cd and MNPs Toxicity in the Ovary

A comprehensive analysis of the available studies indicates that Cd and MPs exhibit molecular convergence: despite their different chemical properties, they activate similar pathways that lead to ovarian damage. The common mechanism of action for both factors is the induction of OS. In both cases, this results in damage to GCs and oocytes and impairs follicular development [25,88,137].

Mitochondrial dysfunction is also a common mechanism of action for Cd and MPs. The consequence is a reduction in the energy function of GCs and oocytes, which may impair folliculogenesis [25,28,154].

Molecular convergence also involves the induction of apoptosis in GCs and oocytes. A common effect of both factors is increased follicular atresia and a deterioration in the quality of the oocyte microenvironment [25,154].

Another key mechanism underlying the combined action of Cd and MPs is the disruption of steroidogenesis. Cd affects the expression of steroidogenic regulators and enzymes, whilst MPs disrupt cholesterol transport to mitochondria and the pathways that regulate granulosa cell function. Consequently, both factors may lead to reduced production of oestrogen and progesterone and to disruption of normal ovarian follicular maturation [25,88,140].

Both groups of pollutants also exhibit genotoxic effects by damaging DNA. The common outcome is an impairment of normal ovarian cell function and the maintenance of tissue homeostasis [25,137,155].

Although few studies have examined direct co-exposure to Cd and MPs, similarities in molecular mechanisms suggest they may have additive or synergistic effects. Both factors activate common pathways involving OS, mitochondrial dysfunction, apoptosis, impaired steroidogenesis and DNA damage, which may lead to impaired ovarian function and reduced reproductive potential [20,25,137,142].

### 5.7. Mechanistic Convergence of Pb and MNPs Toxicity in the Ovary

Combined exposure to Pb and MPs may pose an important toxicological concern due to the potential convergence of their effects on key pathophysiological processes in the ovary. Available experimental evidence indicates that co-exposure to these contaminants may aggravate ovarian injury through interconnected mechanisms involving OS, mitochondrial dysfunction, ER stress, activation of cell-death pathways, and disruption of follicular and steroidogenic function. In this context, the combined effects of Pb and MPs may reflect a toxicological interaction in which the presence of both contaminants leads to convergence on common molecular pathways and potentially increases the severity of ovarian damage compared with individual exposures [142].

OS appears to be one of the most important mechanisms underlying the combined toxicity of Pb and MPs. In a mouse model of combined exposure to PS-MPs and Pb, co-exposure increased ovarian lipid peroxidation and impaired antioxidant defence, as evidenced by elevated malondialdehyde levels and reduced SOD activity. Treatment with N-acetylcysteine attenuated ovarian injury, supporting the involvement of OS in the toxicity associated with combined exposure [142]. These findings suggest that the simultaneous presence of Pb and PS-MPs may intensify redox imbalance in ovarian tissue and promote subsequent cellular damage [142].

ER stress represents another important mechanism involved in the combined toxicity of Pb and MPs. Excessive OS and cellular injury may disrupt protein-folding homeostasis and activate the unfolded protein response. In the ovary, combined exposure to Pb and PS-MPs was associated with activation of the PERK/eIF2α signalling pathway and increased apoptosis. Pharmacological modulation of ER stress reduced ovarian injury, supporting the involvement of this pathway in the response to combined exposure [142]. These findings indicate that ER stress may constitute an important molecular link between oxidative damage and apoptosis following combined Pb–MP exposure [142].

Mitochondrial dysfunction may further contribute to the aggravation of ovarian injury caused by combined Pb and MP exposure. Mitochondria are central regulators of cellular energy metabolism and redox homeostasis. Their impairment may increase ROS production, reduce cellular energy availability, and promote the activation of cell death pathways. In the context of combined exposure, Pb- and MP-induced OS may contribute to mitochondrial dysfunction, which may subsequently amplify ROS generation and cellular stress. This interaction may create a self-reinforcing cycle involving OS, mitochondrial injury, and ER stress. However, direct evidence specifically demonstrating mitochondrial dysfunction as a mechanism of combined Pb–MP toxicity in the ovary remains more limited than the evidence supporting OS and ER stress [142].

Apoptosis represents an important downstream consequence of the convergent cellular stress induced by combined exposure. In the combined Pb–PS-MP exposure model, increased ovarian apoptosis was associated with activation of ER stress and PERK/eIF2α signalling [142]. The loss of ovarian cells, particularly GCs, may impair follicular development, oocyte support, steroidogenesis, and maintenance of the ovarian microenvironment. Therefore, the convergence of OS, mitochondrial dysfunction, and ER stress may ultimately lead to increased apoptotic cell death and structural damage in ovarian tissue [142].

Combined exposure to Pb and MP may also impair follicular homeostasis and steroidogenic function. In the experimental model of combined exposure, co-exposure aggravated ovarian histopathological changes and altered sex hormone levels compared with individual exposures [142]. These effects may result from the combined impact of oxidative and cellular injury on GCs and follicular structures. Consequently, co-exposure may disrupt follicular development and steroidogenic processes, potentially impairing ovarian endocrine and reproductive function [142].

Inflammatory signalling may represent an additional mechanism contributing to the effects of combined Pb and MP exposure. Oxidative damage, organelle dysfunction, and cell death may promote the activation of inflammatory responses within ovarian tissue. However, compared with OS, ER stress, and apoptosis, the specific contribution of shared inflammatory pathways to combined Pb–MP ovarian toxicity remains insufficiently characterised [142].

Overall, the available evidence supports a model in which combined exposure to Pb and MP may induce interconnected oxidative, mitochondrial, ER, and cell death responses in the ovary. A proposed mechanistic sequence is: combined Pb and MP exposure → increased ROS production and redox imbalance → mitochondrial and ER dysfunction → activation of PERK/eIF2α-associated stress signalling → apoptosis of ovarian cells, particularly GCs → impaired follicular function and steroidogenesis. Experimental evidence indicates that combined exposure to Pb and PS-MPs can aggravate ovarian toxicity and increase ovarian Pb accumulation compared with individual exposures [17]. Nevertheless, the terms “synergistic” and “synergy” should be used cautiously unless formal dose–response interaction analyses have been performed. At present, the available evidence more strongly supports the concepts of aggravated combined toxicity and molecular convergence than definitive toxicological synergy.

### 5.8. Mechanistic Convergence of As and MNPs Toxicity in the Ovary

Direct experimental evidence regarding ovarian toxicity following combined exposure to As and plastic particles is currently confined to a limited number of aquatic models, supplemented by mechanistic insights from non-ovarian biological systems. Trivalent inorganic As [As(III)] and MPs exhibit significant mechanistic convergence, identifying biological pathways centred on membrane transporter inhibition, intracellular toxicant retention, oxidative genomic damage, disruption of steroidogenesis, and impaired follicular dynamics.

Direct in vivo evidence in adult female zebrafish demonstrates that co-exposure to 5-μm PS-MPs and As (III) leads to a significantly reduced gonadosomatic index, an abnormal shift in oocyte-stage distribution characterised by a reduced proportion of primary-growth oocytes and an increased proportion of mature oocytes, and marked endocrine disruption evidenced by reduced testosterone levels and an elevated E2-to-testosterone ratio [156]. Untargeted metabolomic profiling further indicates that combined exposure alters distinct ovarian metabolic pathways—over half of which are specific to the co-exposure condition—disrupting aminoacyl-tRNA biosynthesis, sphingolipid, linoleic acid, and nucleotide-sugar metabolism associated with inflammatory and steroidogenic stress [156]. Although co-exposure unexpectedly increased spawning rates, it severely compromised overall reproductive success, resulting in high early offspring mortality and reduced hatching rates [156]. These findings confirm that MPs modify the ovarian response to As, even though joint exposure does not yield uniformly synergistic outcomes across all measured physiological endpoints [156,157].

Evidence from diverse cellular and tissue models indicates that As–MNPs interactions depend strongly on particle dimension, surface properties, and exposure concentrations [158,159,160,161]. NPs (<100 nm) significantly enhance intracellular As bioaccumulation, disrupt membrane fluidity, alter the actin cytoskeleton, and amplify cytotoxic responses to a greater extent than micrometre-scale particles (>1 μm) [158,160,161]. Mechanistically, smaller plastic particles physically impair cellular efflux by inhibiting ATP-binding cassette (ABC) transporters, including P-glycoprotein and multidrug resistance-associated proteins that export As–glutathione conjugates [160,161]. This transporter dysfunction increases the intracellular retention of As, promoting mitochondrial membrane depolarisation, severe ROS generation, oxidative DNA damage, caspase activation, and accelerated cell death [159,160].

When evaluated independently, As and MPs display substantial mechanistic and phenotypic convergence in both mammalian and aquatic ovarian models [20,115,116,152,162,163]. Both pollutants are linked to follicular atresia, depletion of the primordial pool, granulosa-cell injury, and an increased risk of primary ovarian insufficiency [20,115,116,162]. While MP-induced ovotoxicity is driven predominantly by ROS-dependent granulosa-cell apoptosis via Wnt/β-catenin signalling and profibrotic pathways [20], As acts through a broader network involving mitochondrial ROS generation, excessive autophagy, and epigenetic dysregulation, including promoter hypermethylation of *SF-1* [115,116,163]. Nevertheless, both toxicant classes converge directly on key steroidogenic transcripts, including *StAR*, *CYP11A*/*cyp11a*, and *CYP19A1*/*cyp19a1a*, identifying steroid hormone biosynthesis and HPG feedback as primary shared molecular targets [115,116,152].

Overall, the available evidence supports a model of mechanistic convergence in which As(III) and MPs affect the ovary through interconnected transport, oxidative, genomic, apoptotic, and steroidogenic pathways. A proposed sequence is: As(III) and/or MP exposure → particle-size-dependent cellular uptake and ABC transporter inhibition → intracellular As retention, redox imbalance, and increased ROS production → mitochondrial dysfunction, oxidative DNA damage, and ER stress → epigenetic and transcriptional repression of steroidogenic genes (*StAR*, *CYP11A1*, *CYP19A1*) → granulosa-cell apoptosis and altered oocyte maturation → follicular atresia and ovarian endocrine dysfunction. However, because direct co-exposure studies in mammalian ovaries remain lacking, these mechanisms should be interpreted as biologically plausible points of convergence rather than definitive proof of toxicological synergy under all environmental conditions. Future research should directly evaluate combined exposure across varying particle dimensions, chemical speciation, and formal dose–response interaction models.

### 5.9. Mechanistic Convergence of Hg and MNPs Toxicity in the Ovary

A comprehensive analysis of the available studies indicates that MPs and Hg exhibit clear molecular convergence, as they activate the same pathogenic pathways leading to ovarian damage. The best-understood common mechanism of action for both pollutants is the induction of OS. Both Hg and MPs lead to excessive ROS production and disruption of redox homeostasis, which serve as starting points for further pathogenic changes [22,28,87,164]. The first of these is mitochondrial dysfunction. In both Hg and MP cases, the mitochondrial apoptotic pathway is initiated [25,87,137,165].

Molecular convergence also involves inhibiting granulosa cell proliferation. Both factors activate DNA damage response mechanisms, induce cell cycle arrest and suppress the expression of genes responsible for cell growth and survival. As a result, the maturation of ovarian follicles is disrupted, and the ovary’s ability to maintain normal reproductive function is impaired [25,124,137].

Another key aspect of their combined effect is the disruption of steroidogenesis. Hg reduces the expression of the StAR protein and cytochrome P450, whilst MPs further disrupt the expression of genes regulating lipid metabolism and cholesterol transport. The result is a reduction in progesterone and oestrogen synthesis, as well as disruption of communication between GCs and the oocyte [124,137,166].

Both groups of pollutants also exhibit genotoxic potential. Excessive ROS production leads to oxidative DNA damage, DNA strand breaks, and disruption of repair mechanisms [25,167].

Although there are still few direct studies on co-exposure of the ovary to Hg and MPs, experimental results shared molecular mechanisms strongly suggest toxic synergy between the two factors and justify the need for further experimental and epidemiological research [25,87,137,141,142].

### 5.10. Comparative Synthesis of Trace Elements and MNPs Convergence

Of the trace elements discussed, Pb and As provide the strongest direct experimental evidence for a convergence of mechanisms of action with MNPs in the reproductive system [142,156]. In the case of Pb, studies using a mouse model of co-exposure to Pb and polystyrene MNPs (PS-MNPs) confirmed that simultaneous exposure drastically increases lipid peroxidation, reduces antioxidant defence and activates ER stress and the PERK/eIF2α signalling pathway, which together lead to increased apoptosis of GCs and oocytes [142]. With regard to As, in vivo studies in adult female Danio rerio have shown that co-exposure to MNPs and As(III) induces significant endocrine disruption (reduced testosterone levels, abnormal E2:testosterone ratio), changes in oocyte distribution, and alters specific ovarian metabolic pathways, including sphingolipid metabolism [156]. For other elements, evidence of convergence is based on logical deduction from an analysis of the overlapping mechanisms of action of individual substances or on reports from non-ovarian models [132,133,134,137,142].

There is a clear difference in the mechanisms by which MNPs interact with essential elements (Zn, Se, Cu, Cr, Mn) compared to toxic and physiologically irrelevant metals (Cd, Pb, As, Hg). Essential elements are characterised by a narrow homeostatic window, within which they perform key functions supporting cellular antioxidant defence by acting as cofactors for critical enzymes such as Cu/Zn-SOD1, GPX1 and MnSOD [31,49,81]. In their case, interactions with MNPs are highly dose-dependent: at physiological concentrations, an adequate supply of these elements (as documented, for example, with nano-Se in studies on the liver and intestines) may potentially mitigate MNP-induced toxicity by restoring the activity of antioxidant enzymes and reducing inflammation [132,133,134]. Conversely, there are no non-toxic doses of heavy metals—elements such as Cd, Pb, As or Hg damage GCs and oocytes regardless of concentration [87]. Their interaction with MNPs leads solely to an exacerbation of cellular stress, whereby both environmental factors jointly increase the production of ROS, deplete cellular glutathione stores, exacerbate mitochondrial dysfunction and cause structural damage [22,25,28,89,137]. Another area of research concerns epigenetic mechanisms; in this case, unlike As, Cd or Cu, the current literature on the effects of Pb and Hg on the ovaries focuses on structural damage, endoplasmic reticulum (ER) stress and direct DNA strand breaks, leaving the epigenetic mechanisms of these two metals as a significant gap requiring further research.

Although all analysed xenobiotics induce OS, their effects on specific biomarkers differ, suggesting distinct pathogenic pathways. Exposure to MNPs, As, and Hg manifests a highly consistent profile: a drastic reduction in reduced GSH levels and an increase in MDA concentration, a marker of membrane lipid peroxidation [24,111,118,123]. Furthermore, MNP and As lead to a reduction in CAT and SOD activity [24,111]. In turn, essential elements are structurally linked to specific enzymatic isoforms, and their deficiency or excess affects specific targets: Zn and Cu determine the activity of cytoplasmic Cu/Zn-SOD1 [31,59], Mn controls mitochondrial MnSOD (critical for oocyte protection during steroidogenesis) [80], while Se plays a key role in maintaining the pool of glutathione peroxidases (including GPX1) neutralising H_2_O_2_ in the follicle microenvironment [49].

A common, overarching factor in the interaction of MNPs with all the elements discussed is the induction of oxidative stress, mitochondrial dysfunction, disruption of local steroidogenesis and apoptosis of granulosa cells, which ultimately results in follicular atresia [20,25,88,135,137]. However, element-specific mechanisms have been identified: As, in combination with nanoplastics, is characterised by physical impairment of intracellular efflux via the inhibition of ABC membrane transporters, which prevents the efflux of toxins from the cell and enhances the internal retention of As [160,161]. Furthermore, As triggers specific epigenetic silencing of steroidogenic genes (including *StAR* and *CYP11A1*) via promoter hypermethylation [116]. Cd induces characteristic cell destruction by triggering enhanced mitophagy and ferroptosis, which, when combined with the antioxidant deficit generated by MNPs, leads to the destruction of the cell membranes of oocytes and granulosa cells [89,90,91]. Pb is strongly associated with the UPR pathway, suggesting that the primary link between oxidative damage and MNP-induced apoptosis in this case is direct endoplasmic reticulum stress [106,142].

Based on the strength of the experimental evidence and the degree of convergence of pathological pathways, the following testable hierarchy of relative toxicological risk to the ovary under conditions of co-exposure can be proposed: Highest risk (directly documented potentiation of toxicity): Co-exposure to MNPs in combination with As and Pb [142,156]. Thanks to direct in vivo evidence confirming increased accumulation of toxins in cells (As) and the activation of ER stress cascades (Pb), these interactions constitute an experimentally verified risk that accelerates ovarian degeneration and reduces the chances of reproduction [142,159,160]. High risk (highly probable additive convergence): Co-exposure to MNPs in combination with Cd and Hg [87,88,124]. Despite the lack of direct co-exposure studies on ovarian tissue, the confirmed ability of Cd to induce potent ferroptosis [90,91] and Hg’s ability to generate destructive DNA strand breaks [25,122,167], combined with the pathogenesis induced by MNPs, unequivocally suggests irreversible and intensified depletion of the ovarian reserve. Modifiable risk (dependent on the homeostatic window): Co-exposure to MNPs and essential elements (Zn, Se, Cu, Cr, Mn). The toxicity of this group is concentration-dependent [10]. An excess of these minerals will synergistically exacerbate the oxidative stress generated by plastics [81,135]. However, at physiological doses, the presence of these trace elements (particularly Zn and Se) has the potential to exert an antagonistic effect, whereby the enhancement of the cellular pool of antioxidant enzymes may effectively prevent apoptosis induced by the presence of MNPs [34,130,133,134].

## 6. Future Perspectives

Most laboratory studies to date have exposed GCs or animal models exclusively to MNPs or individual heavy metals. Future studies should investigate their effects on the ovary under conditions of co-exposure, which should be the focus of future research. This will enable the degree of contamination of the ovarian microenvironment to be determined, which may, in the future, constitute a significant element in the diagnosis of idiopathic infertility and ovarian dysfunction, as well as in the assessment of risks to ovarian reserve. The findings of research into the toxic convergence of MNPs and metals should provide a scientific basis for public health authorities to rigorously restrict the use of plastics in food and medicine packaging, as well as to review the permissible concentration limits for MPs in the environment regarding women’s reproductive health. Some recent in vivo and in vitro studies have used doses of MNPs or trace elements that significantly exceed actual human exposure levels, which may mean that the effects of these particles do not reflect actual human exposure levels. Future studies should take these discrepancies into account in order to distinguish between mechanisms relevant from an environmental perspective and those that occur solely in response to high or acute doses, thereby enabling an assessment of the actual impact of these particles on ovarian function. Furthermore, future research should also focus on exploring potential methods to prevent the adverse effects of MNPs and trace elements by mitigating the pro-oxidative loop in the ovarian microenvironment in patients with high occupational or environmental exposure, as well as on targeted mineral and antioxidant supplementation.

An important, yet insufficiently explored, mechanistic pathway is transgenerational effects. Current evidence indicates that paternal exposure to Cd induces epigenetic changes in sperm that are passed on to female offspring, causing impaired hormone synthesis in granulosa cells in daughters without their direct exposure to the metal. Similar multigenerational effects on reproductive performance have been observed in animal models with co-exposure to MNP and As, resulting in high mortality of offspring during early development. The phenomenon of intra- and intergenerational transmission of ovarian epigenetic damage for most trace elements remains largely unknown and represents a key priority for future research.

## 7. Limitations

This article, which is a literature review, analyses the role of trace elements in ovarian function and in associated disorders. For this reason, the article is subject to certain methodological limitations—despite the broad scope of the data discussed, it is not a systematic review; the absence of a systematic review approach limits the ability to assess the reliability of the conclusions drawn from individual studies. The information contained in the individual sections is derived from cellular studies, animal models, observational studies in humans and interventional studies. Differences in study designs, exposure methods, duration and measurement techniques mean that the results of individual studies may be inconsistent; however, their inclusion is essential to illustrate the broad perspective of the issue under investigation. The chapter discussing the convergence of the mechanisms of action of trace elements and magnetic nanoparticles (MNPs) draws primarily on studies demonstrating similar mechanisms of action for these substances; this highlights a gap in the literature, although the speculative nature of this section should also be noted, particularly in the subsections on the convergence of MNPs and Cu, Cr, Mn and Se.

## 8. Conclusions

Essential trace elements, such as Zn, Se, Cu, Cr and Mn, play irreplaceable physiological roles as enzyme cofactors and transcriptional regulators, which are essential for normal folliculogenesis, steroidogenesis and the meiotic maturation of oocytes. However, their biological action is confined to a narrow homeostatic window—both intracellular deficiency and excessive accumulation disrupt the redox state, alter HPG axis signalling and contribute to ovarian dysfunction and metabolic disorders in PCOS. The effects of toxic trace elements, including Cd, Pb, As and Hg, differ; they exhibit non-threshold cytotoxic, genotoxic, and endocrine-disrupting effects in ovarian tissue. Exposure to these xenobiotics reduces the viability of granulosa and theca cells, induces ferroptosis, causes endoplasmic reticulum stress and silences key steroidogenic enzymes via promoter hypermethylation, leading to accelerated depletion of the ovarian reserve and an increased risk of premature ovarian failure.

At the same time, environmental MNPs overcome biological barriers and accumulate in the ovarian microenvironment and in follicular fluid, where they stimulate ROS-dependent apoptosis of granulosa cells, induce pyroptosis via the NLRP3/caspase-1 pathway, disrupt cytoskeletal integrity and play a role in fibrotic tissue remodelling. Importantly, although MNPs and heavy metals differ in their chemical properties, they exhibit marked molecular convergence on common pathogenic pathways. Concurrent exposure increases intracellular retention of toxins by inhibiting ABC transporters, exacerbates mitochondrial depolarisation, activates PERK/eIF2α stress signalling, and accelerates programmed cell death. This convergent pathophysiology suggests that co-exposure may lead to additive or synergistic effects, amplifying the risk to reproductive health beyond the thresholds established for individual substances. Consequently, these findings highlight the urgent need to develop multi-component risk assessment models in female reproductive toxicology and provide a scientific basis for implementing stricter regulatory standards and targeted antioxidant strategies to protect ovarian function.

## Data Availability

No new data were created or analyzed in this study. Data sharing is not applicable to this article.

## Abbreviations

The following abbreviations are used in this manuscript:

ABC	ATP-binding cassette
ACSL4	Acyl-CoA synthetase
AMH	Anti-Müllerian hormone
AMPK	AMP-activated protein kinase
As	Arsenic
As(III)	Trivalent inorganic arsenic
ATOX1	Antioxidant 1 copper chaperone
ATP7A	ATPase copper transporting alpha
ATP7B	ATPase copper transporting beta
CAT	Catalase
CCS	Copper chaperone for superoxide dismutase
Cd	Cadmium
CDK1	Cyclin-dependent kinase 1
COX17	Cytochrome c oxidase copper chaperone 17
COX2	Cyclooxygenase 2
Cr	Chromium
Cr(VI)	Hexavalent chromium
CTR1	Copper transporter 1
Cu	Copper
DAAM-1	Disheveled-associated activator of morphogenesis
E2	17β-estradiol
ER	Endoplasmic reticulum
FSH	Follicle-stimulating hormone
FTH1	Ferritin heavy chain 1
GCs	Granulosa cells
GnRH	Gonadotropin-releasing hormone
GPX	Glutathione peroxidase
Hg	Mercury
HPG	Hypothalamic–pituitary–gonadal axis
HSD	Hydroxysteroid dehydrogenase
IL	Interleukine
IVF	In vitro fertilisation
LH	Luteinising hormone
Mn	Manganese
MNPs	Micro- and nano-plastics
MPs	Microplastics
NADPH	Nicotinamide adenine dinucleotide phosphate (reduced form)
NF-κB	Nuclear factor kappa B
NLRP3	NLR family pyrin domain-containing 3
NPs	Nanoplastics
Nrf2/Keap1	Nuclear factor erythroid 2-related factor 2/Kelch-like ECH-associated protein 1
OS	Oxidative stress
Pb	Lead
PCNA	Proliferating cell nuclear antigen
PCOS	Polycystic ovary syndrome
PERK-eIF2α-ATF4-CHOP	Protein kinase RNA-like endoplasmic reticulum kinase–eukaryotic translation initiation factor 2 alpha–Activating Transcription Factor 4–C/EBP Homologous Protein
PI3K	Phosphoinositide 3-kinase
pSMAD2/3	Phosphorylated SMAD2/3
PS-MPs	Polystyrene microplastics
ROS	Reactive oxygen species
Se	Selenium
SF-1	Steroidogenic factor 1
SOD	Superoxide dismutase
StAR	Steroidogenic acute regulatory protein
TGF-β	Transforming growth factor beta
Wnt	Wingless-related integration site
ZIP	Zrt- and Irt-like protein (zinc importer)
Zn	Zinc
ZnO-NPs	Zinc oxide nanoparticles
ZnT	Zinc transporter
